# Long non-coding RNA TCL6 enhances preferential toxicity of paclitaxel to renal cell carcinoma cells

**DOI:** 10.7150/jca.32552

**Published:** 2020-01-01

**Authors:** Zhizhao Chen, Quan Zhuang, Ke Cheng, Yingzi Ming, Yujun Zhao, Qifa Ye, Sheng Zhang

**Affiliations:** 1 The Third Xiangya Hospital of Central South University, Changsha, China;; 2 Wuhan University, Zhongnan Hospital of Wuhan University, Institute of Hepatobiliary Diseases of Wuhan University, Transplant Center of Wuhan University, Hubei Key Laboratory of Medical Technology on Transplantation, Wuhan Hubei, China.

**Keywords:** Renal cell carcinoma, Apoptosis, Paclitaxel, Chemotherapy.

## Abstract

**Background:** Recent findings have shown long non-coding RNAs (lncRNAs) are dysregulated in a variety of cancer cells. In this report, we investigate the effect of T-cell leukemia lymphoma 6 (TCL6) on paclitaxel (PTX)-induced apoptosis in Renal cell carcinoma (RCC) cells.

**Methods:** Expression levels of TCL6 in RCC tissues were analyzed via The Cancer Genome Atlas (TCGA) and Gene Expression Omnibus (GEO) datasets. Fluorescence in situ hybridization (FISH) was performed to detect the expression of TCL6 in RCC tissues and cells. Two pairs of cell lines were used: TCL6-silenced 786-O cell line and scrambled 786-O cell line, TCL6-overexpressed Caki-1 cell line and Caki-1 scrambled cell line. Cell viability was detected using the MTT assay. Apoptosis was examined by flow cemetery. Dual reporter gene assay was performed to confirm the direct downstream target miRNA of TCL6.

**Results:** Based on RNA sequencing expression data of RCC tissues from TCGA and GEO datasets, the expression deficiency of TCL6 was observed in RCC tissues. Low level of TCL6 was associated with worse overall and disease-free survival of RCC patients. The FISH showed similar results with low expression of TCL6 in RCC tissues and cells. After PTX treatment, a time-dependent decrease in cell viability was observed in TCL6-overexpressed RCC cells and an increase in cell viability was observed in TCL6-silenced cells compared to control cells. Apoptosis induced by PTX was significantly increased in TCL6-overexpressed cells. Inhibition of TCL6 showed a significant decrease in apoptosis. Furthermore, luciferase reporter assay revealed that TCL6 is a direct target gene of miR-221.

**Conclusions:** TCL6 effectively sensitizes RCC to PTX mainly through downregulation of miR-221. Our results suggest that PTX combined with TCL6 might be a potentially more effective chemotherapeutic approach for renal cancer.

## Introduction

RCC is an epithelial tumor derived from the proximal tubules of the nephrons and it is the most common primary tumor of the renal parenchyma, accounting for approximately 90% of kidney neoplasms and 3% of all malignancies 1, 2. The most common histological RCC type is the clear cell RCC, which is estimated as 80% of all patients 3. The currently available therapeutic options including chemotherapy, radiotherapy, targeted therapy, and immunotherapy are ineffective and the clinical management of advanced RCC is still challenging 4, 5. A primary cause of the resistance and subsequent treatment failure is RCC intra-tumoral heterogeneity 6-8. Targeting common elements between different molecular/genetic subclasses of RCC may represent a potential therapeutic strategy.

LncRNAs are a highly heterogeneous group of untranslated RNA molecules with over 200 nucleotides but lack open reading frames 9. LncRNAs are frequently aberrantly expressed in multiple cancers having regulatory roles in fundamental biological processes, which function as oncogenes or suppressor genes in tumor initiation and progression. Recent studies of RCC have reported lncRNA expression profiles by gene microarray analysis and identified the roles of specific lncRNAs such as HOTAIR 10, MALAT1 11, GAS5 [12]and LINC00961 13. However, the detail function of lncRNAs in RCC remains largely unknown. TCL6, also named TNG1 or TNG2, located on 14q32.13. A recent study demonstrated that decreased expression of TCL6 was associated with poor prognosis in patients with RCC 14.

PTX is an effective mitotic inhibitor and apoptosis inducer. It has been widely used in chemotherapy for multiple cancers 15. How to enhance the sensitivity of renal cancer cells to PTX is still a problem that needs to be solved.

TCGA, a public available platform with more than 30 types of cancer from 11,000 patients at least and their clinicopathological information, has been widely applied by large numbers of researches to explore the genetic basis of tumor according to the high-throughput sequencing 16. In the current study, we found the expression deficiency of TCL6 was observed in the RCC tissues through analysis of TCGA and GEO databases. Low level of TCL6 was associated with worse overall and disease-free survival of RCC patients. These findings hint that TCL6 plays an important role in the progression of RCC. We aimed to elucidate the potential mechanisms of TCL6 modulating survival of cancer cells after PTX treatment.

## Materials and methods

### TCGA dataset of RCC clinical samples

The online data of TCL6 expression level, clinical and prognosis characteristics were analyzed by Gene Expression Profiling Interactive Analysis (GEPIA) (http://gepia.cancer-pku.cn/detail.php###), The Atlas of Noncoding RNAs in Cancer (TANRIC) platforms (https://ibl.mdanderson.org/tanric/_design/basic /query.html) and GEO datasets. GEPIA and TANRIC are web servers for analyzing the RNA sequencing expression data from the TCGA database. Search strategies were as previous report 17, 18.

10 cases of human RCC tissues and adjacent normal renal tissues were formalin-fixed and paraffin-embedded. All specimens were collected from the Third Xiangya Hospital of Central South University.

### Cell culture

Human renal proximal tubule epithelial cell line (HK-2) and RCC cell lines (OS-RC-2, KC, ACHN, 786-O, and Caki-1) were purchased from American Type Culture Collection (ATCC). HK-2 cells were cultured in DMEM/F12 containing 10% FBS. 786-O cells were cultured in 1640 medium supplemented with 10% FBS at 37˚C with 5% CO_2_ in a humidified atmosphere. OS-RC-2, KC, ACHN, and Caki-1 cells were cultured in DMEM/10% FBS medium.

### Fluorescence in situ hybridization (FISH)

All specimens and cell lines were subjected to FISH for TCL6 expression using the FISH kit (C10910; Ribobio, Guangzhou, China). The red FISH Probe of TCL6 (Lnc1CM001, 5'-CTATCCATTCAGCATCAGAGA-3'), U6 (Lnc110101, internal reference) and 18S (Lnc110201) were purchased from Ribobio (Guangzhou, China). Briefly, pre-hybridization buffer was added to unstained RCC tissue sections for 30 min. And then TCL6 FISH Probe Mix was added to each section and left overnight. Nuclear was stained by blue DAPI incubation at room temperature for 10 min. Cells (5×10^4^/well) were seeded in 24-well plate and then fixed by 4% paraformaldehyde. 200 µL of pre-hybridization solution was added in each well for 30 min. 200 μL of hybridization solution was then added, and incubation at room temperature overnight. Fluorescence microscopy was used to capture images.

### TCL6 siRNA and overexpression plasmid transfection

TCL6 was inserted the pcDNA3.1 plasmid (Invitrogen, Carlsbad, CA, USA) by Yingrun Company (Changsha, China). Knockdown of TCL6 was performed using short interfering (si) RNAs provided by Guangzhou Ribobio Co., Ltd. (Guangzhou, China). The siRNA sequences were as follows: si1-TCL6: 5'-CCTTAACCTTGGCACAACA-3', si2-TCL6 forward: 5'-ACTCACAG AGGAGCAGAAT-3', and si3-TCL6 forward: 5'-GCAATCAGAAACAGAAAGT-3'. scrambled control siRNAs were synthesized by Ribobio (Guangzhou, China). Cells were plated in 6-well plates. Transfection was performed when cells reached 80% confluence using 10 µl Lipofectamine® 2000 (Thermo Fisher Scientific). To validate the efficiency of transfection, lncRNA expression was examined by fluorescence microscope and qRT-PCR.

### Quantitative real-time RT-PCR

Total RNA from cells and tissues were extracted with TRIzol reagent (Invitrogen; Thermo Fisher Scientific, Inc) according to the manufacturer's protocol. SuperScript II Reverse Transcriptase kit (Invitrogen Life Technologies) was used to generate cDNA. Quantitative real-time PCR analyses were performed according to previous study 19. Samples were compared using the relative CT method, where the relative expression of TCL6 was normalized to that of GAPDH. U6 served as internal control for the quantification of miR-221. The primers were synthesized by Ribobio (Guangzhou, China) and the sequences as follows: TCL6 forward, 5'-CTATCCATTCAGCATCAGAGA-3' and reverse, 5'-CACATACTCACGCATCCTT-3' (Ribobio, Guangzhou, China). GAPDH forward, 5'-ATGACATCAAGAAGGTGG TG-3' and reverse, 5'-CATACCAGGAAATGAGCTTG-3' (Ribobio, Guangzhou, China).

### Cell proliferation assay

The MTT assay was used to measure cell proliferation after PXT (Beyotime, Shanghai, China) treatment. Cells (5×10^3^) were seeded in 96-well plates and cultured for 24, 48, 72 and 96 h, and then the supernatant was removed and MTT (5 mg/ml, 20 μl, Beyotime, Shanghai, China) was added to each well at 37˚C. After 4 h, 100 μl DMSO was added to each well. The OD value was determined at 490 nm by a microplate reader and used to construct a growth curve to assess cell proliferation.

### Apoptosis analysis

Apoptosis was evaluated using Annexin V-FITC kit (Beyotime, Shanghai, China), Cells (2 × 10^6^) were seeded in T25 cell culture flask and incubated for 48 h. Cells were suspended in 195 μL Annexin V-FITC binding buffer. 5μL of Annexin V-FITC and 10 μL of propidium iodide (PI) were added and then incubated for 20 min at 25℃ in the darkroom. The percentage of apoptotic cells was evaluated using a flow cytometer.

### Dual-luciferase reporter assays

Luciferase reporter assays were performed using the pmirGLO-TCL6-Luc vector. HEK293 cells (ATCC, Manassas, VA, USA) were co-transfected with pmirGLO-TCL6-Luc vector (1 µg) and miR-221-mimics or NC-mimics (40 pmol). Cells were harvested 48 h after transfection and analyzed with the dual-luciferase reporter gene assay kit E1910 (Promega). The firefly luciferase values were normalized to Renilla luciferase values as an internal control.

### Statistical analysis

All experiments were repeated at least three times. Statistical analyses were carried out using SPSS 19.0 software (SPSS, Chicago, IL, USA). The results were assessed by Student's t-test or one-way analysis of variance. All statistical tests were two-sided, and a P-value of < 0.05 was considered to indicate a statistically significant difference.

## Results

### The expression deficiency of TCL6 was observed in RCC tissues

**Figure 1:**
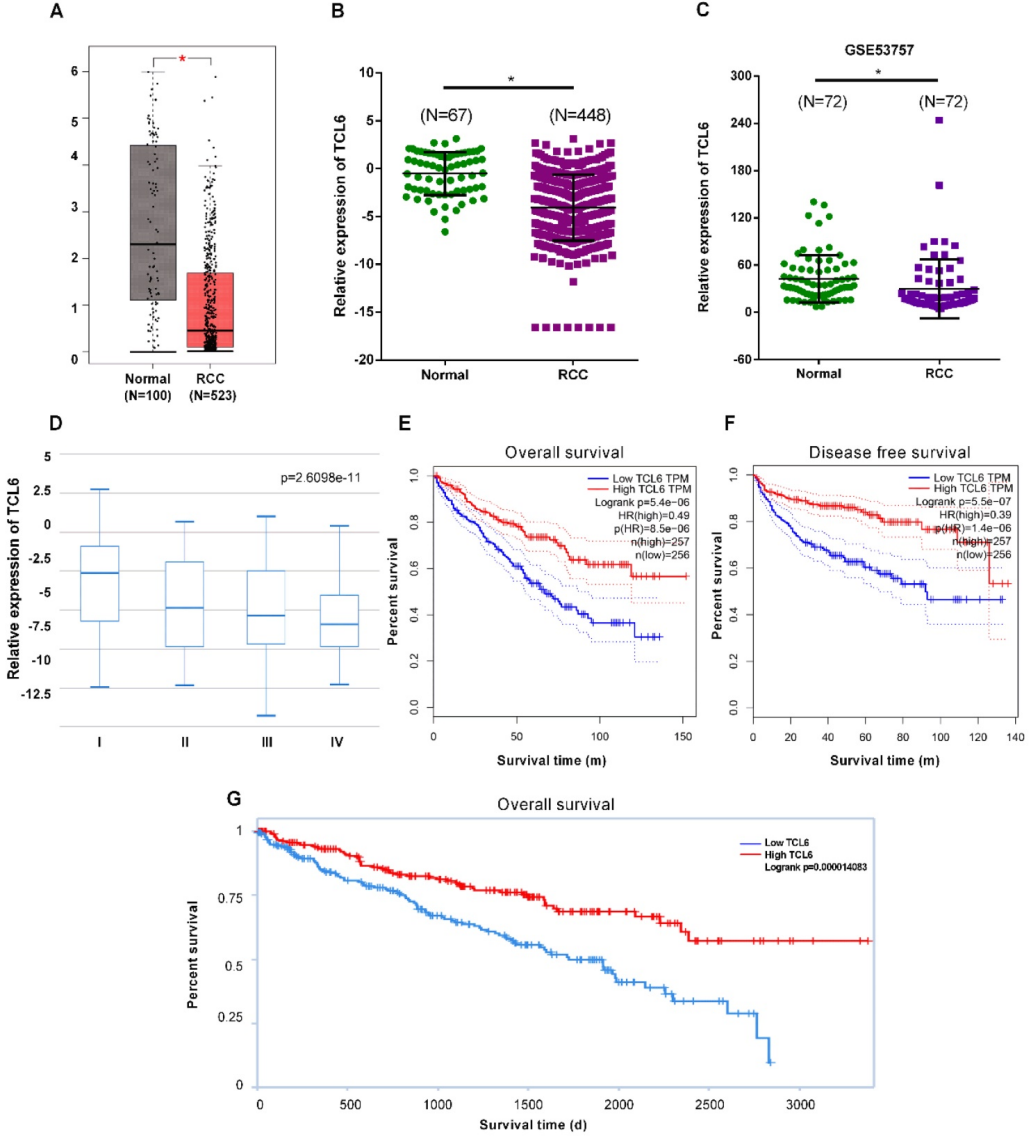
The downregulation of TCL6 expression was associated with poor prognosis of patients. A. The expression of TCL6 was decreased in RCC tissues (523 cases) compared to the normal tissues (100 cases), which of data was collected from the TCGA database and analyzed by GEPIA platform. B. The TCL6 expression data of RCC (n=448) and normal (n=67) tissues were further analyzed by online TANRIC platform. C. We further analyzed the expression levels of TCL6 in seventy-two RCC and match normal tissues obtained from the GEO database (GSE53757dataset). D. The expression of TCL6 gradually decreased among the different clinical stages of renal cancer (by one-way analysis of variance). Data are shown as means ± SD. Kaplan-Meier curves showed the patient overall survival (E) and disease-free survival (F) of the high TCL6 expression and low TCL6 expression. These graphs were conducted by TANRIC platform. Dotted line: the 95% confidence interval (CI). G. Low level of TCL6 expression was associated with poor overall survival of patients with renal cancer. *P<0.05.

To elucidate expression levels of TCL6 in RCC tumorigenesis, the GEPIA platform was performed to comprehensively analyze the TCL6 in 523 cases of RCC tissues and 100 cases of normal renal tissues. The results revealed that TCL6 was significantly decreased in cancer versus normal tissues (Fig.1A). These data indicate that TCL6 may regulate tumorigenesis and development of RCC. We next analyzed the expression levels of TCL6 using TANRIC platform. The results of the analysis showed that the TCL6 was significantly down-regulated in 448 cases of RCC tissues as compared to 67 cases of normal renal tissues (Fig.1B). We analyzed the TCL6 expression in 72 cases of fresh RCC and adjacent normal tissues from GEO dataset #GSE53757. The data indicate that the TCL6 expression was lower in RCC tissues than adjacent normal tissues (Fig.1C). And TCL6 expression is progressively reduced in advancing clinical stages of renal cancer (Fig.1D). Low level of TCL6 was associated with worse overall (Fig.1E, G) and disease free survival (Fig.1F) of RCC patients.

### TCL6 inhibits proliferation after PTX treatment

**Figure 2:**
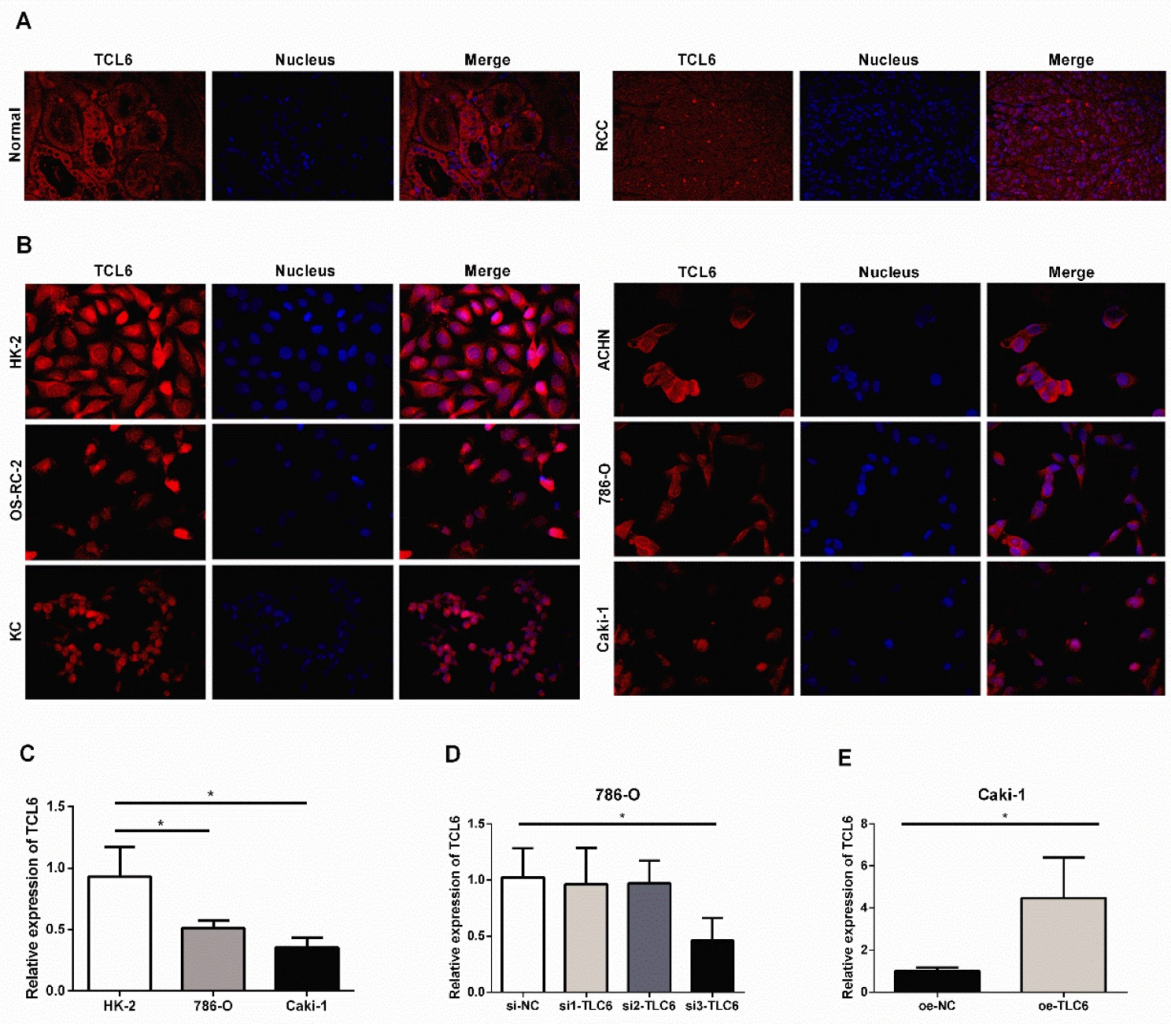
The relative expression levels of TCL6 in RCC tissues cells. A. Confocal FISH images exhibited expression and localization of TCL6 in RCC and adjacent normal renal tissues. TCL6 (red signal) was mainly located in the cytoplasm. Blue DAPI for nucleus staining. B. The levels of TCL6 expression was detected by FISH in HK-2 cells and 5 RCC cell lines. There were less red signals in 5 RCC cell lines compared to HK-2 cells. C. The basal expression of TCL6 in HK-2, 786-O, and Caki-1 cells was evaluated by qRT-PCR. D. 786-O cells were transfected with TCL6 siRNA1, siRNA2, siRNA3, and NC siRNA, respectively. qRT-PCR revealed the expression levels of TCL6 were significantly reduced by treatment with TCL6 siRNA 3. E. The expression levels of TCL6 were elevated in Caki-1 cells transfected with TCL6 overexpression plasmids compared to those cells were treated with the negative control plasmids. Data are shown as means ± SD (n=3). FISH: fluorescence in situ hybridization; *P<0.05.

We further examined the expression level of TCL6 in human RCC tissues and cells using FISH. The FISH showed similar results with low expression of TCL6 in RCC tissues compared to the adjacent normal renal tissues (Fig.2A). LncRNA TCL6 was mainly located in the cytoplasm. We further detected the expression of TCL6 in human renal proximal tubule epithelial cell line (HK-2) and RCC cell lines (OS-RC-2, KC, ACHN, 786-O, and Caki-1). TCL6 was down-regulated in all RCC cell lines compared to the HK-2 cells as evidenced by FISH (Fig.2B). 786-O and Caki-1 cells were used for further experiments based on their showed lower expression of TCL6 compared to OS-RC-2, KC, ACHN cells. As shown in Fig.2C, the expression of TCL6 was downregulated in 786-O and Caki-1 cells compared to HK-2 cells as evidenced by qRT-PCR.

Our findings and a previous study 14 suggested that TCL6 plays an important role in the progression of RCC. But the impact of TCL6 on RCC cell PTX chemotherapy is unclear. To investigate this issue, we modify the expression level of TCL6 and analyzed its impacts on the cell viability of 786-O and Caki-1 cells with PTX.

**Figure 3:**
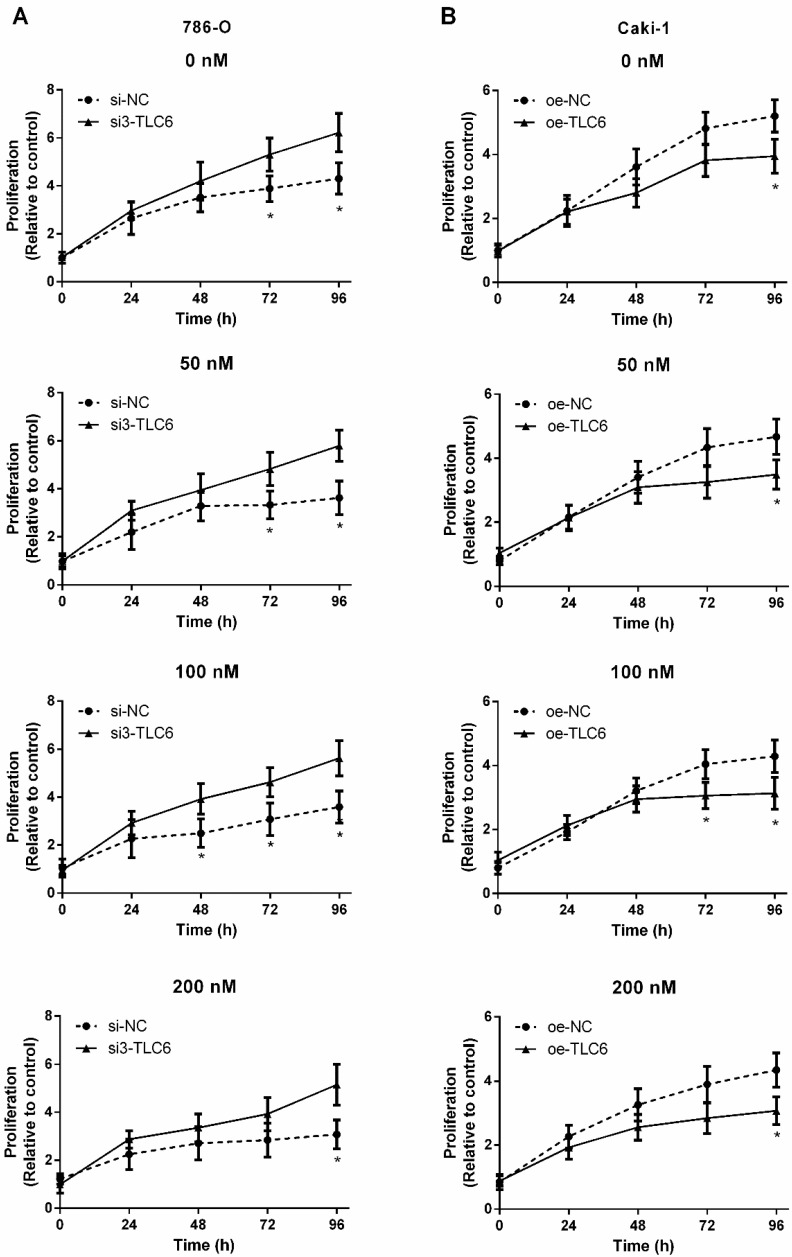
TCL6 expression is correlated with sensitization of RCC cells to PTX. The indicated concentrations of PTX were treated to two kinds of renal cancer cells for 0, 24, 48, 72, and 96 h. A. The proliferation of TCL6-inhibited 786-O cells were significantly increased as compared to those 786-O cells treated with scramble control siRNAs (si-NC) with different levels of PTX concentration (0, 50, 100, 200 nM) at 96 h. B. After incubation for 96 h, the proliferation of TCL6-increased Caki-1 cells was significantly lower than those cells treated with empty pcDNA3.1 plasmid (oe-NC) with different concentrations of PTX. si-NC: 786-O cells treated with scrambled control siRNAs; si3-TCL6: 786-O cells treated with si3-TCL6 siRNAs; oe-NC: Caki-1 cells treated with empty pcDNA3.1 plasmid. oe-TCL6: Caki-1 cells treated with TCL6 overexpressing plasmids. Data are shown as means ± SD. *P<0.05.

Three siRNAs target TCL6 were transfected into 786-O cells, and the third one revealed the best knockdown effect as evidenced by qRT-PCR (Fig.2B). The results of qRT-PCR show that TCL6 expression in Caki-1 cells was notably increased after transfection with TCL6 overexpressing plasmid (Fig.2C). The cell viability was analyzed by MTT assay after being treated with various concentrations of PTX. As shown in Fig.3A, the cytotoxicity of PTX in TCL6 deficient 786-O cells was reduced compared to control 786-O cells. Suppression of cell growth by PTX on TCL6 overexpressing Caki-1 cells was more significant than that of control Caki-1 cells (Fig.3B), indicating more severe PTX-induced cytotoxicity to TCL6-enriched cells.

### TCL6 induces apoptosis after PTX treatment

**Figure 4:**
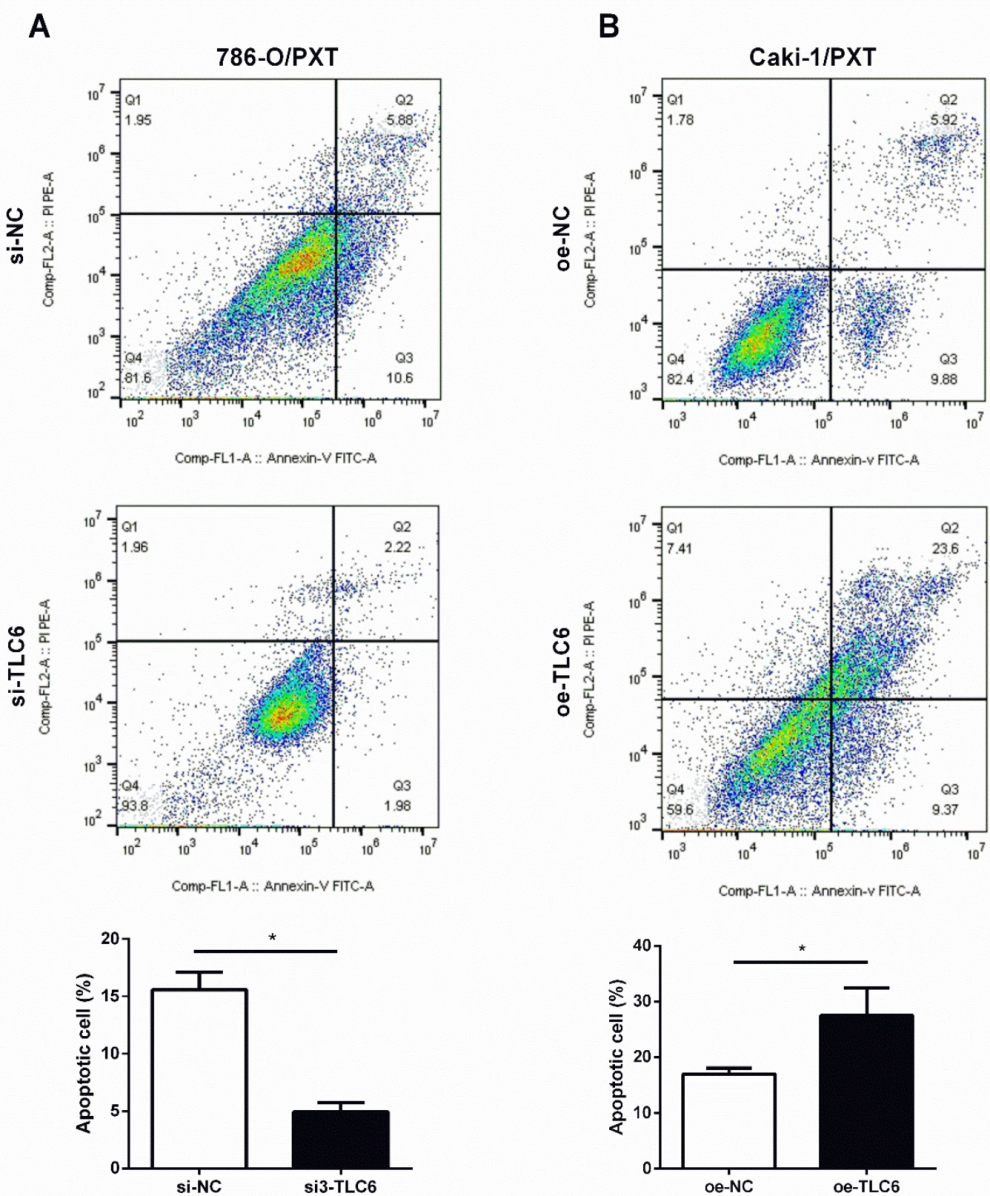
Modulation of PTX cytotoxicity by overexpression (oe-TCL-6) or inhibition (si3-TLC6) of TLC6. A. The apoptosis assays of 786-O cells were performed with (si3-TCL6) or without si3-TCL6 siRNAs (si-NC) under PTX treatments. B. Inversely, up-regulation of TCL6 decreased the apoptosis of Caki-1 cells compared to control cells (oe-NC). Data are shown as means ± SD (n=3). *P<0.05.

PTX is an effective apoptosis inducer. To further investigate whether TCL6 exerts synergistic cytotoxicity effects with PTX in RCC cells, the apoptosis of 786-O and Caki-1 cells was evaluated by flow cytometer. Down-regulation of TCL6 expression in 786-O cells caused an inhibition of apoptosis compared with scrambled control 786-O (Fig.4A). Moreover, comparing to scrambled control Caki-1 cells, the apoptotic cell death apoptosis was significantly induced by overexpression of TCL6 (Fig.4B).

### TCL6 sensitizes RCC cells to PTX through sponging miR-221

**Figure 5:**
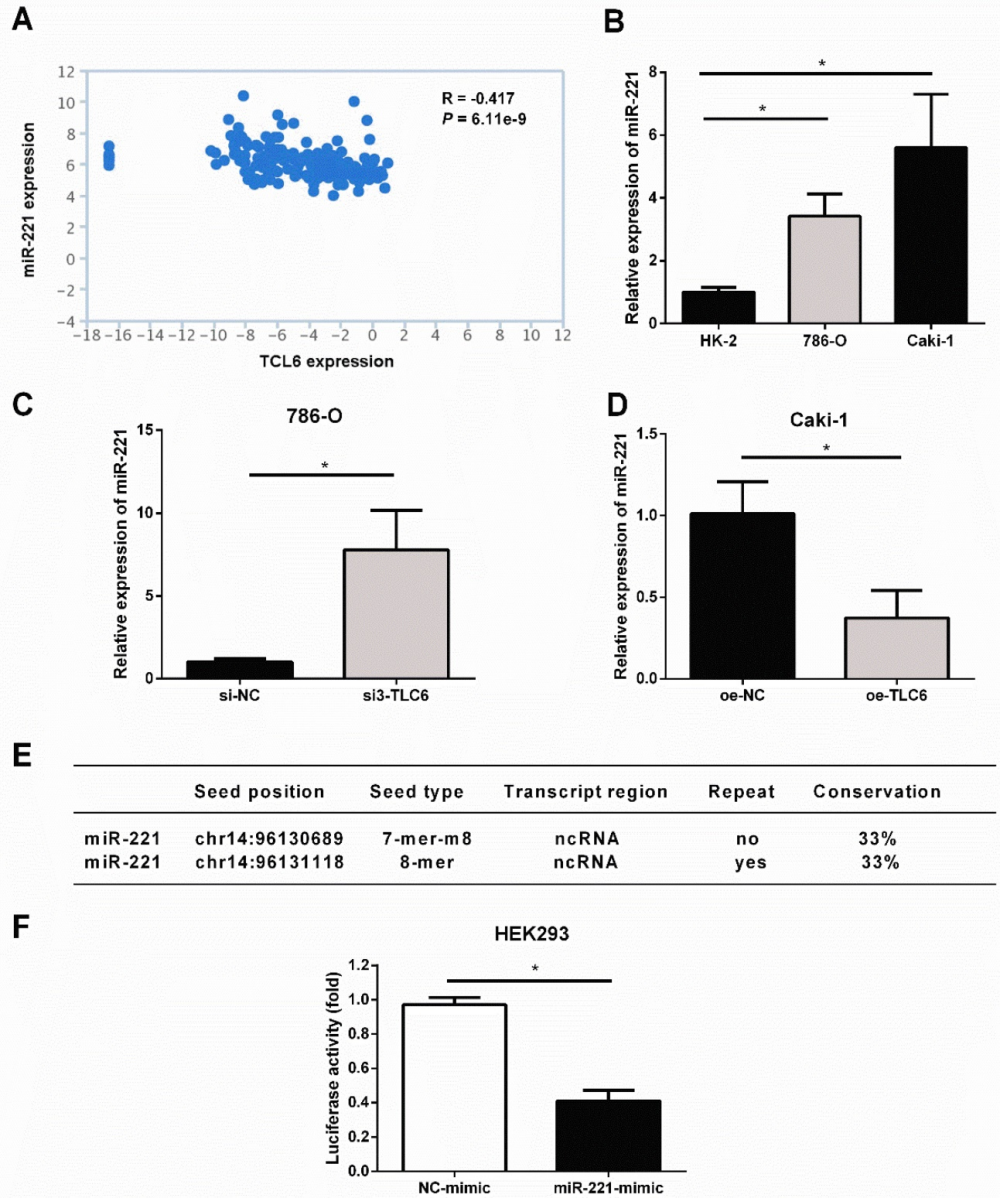
TCL6 sensitizes RCC cells to PTX through sponging miR-221. A. The level of miR-221 was negatively correlated with TCL6 expression in renal cancer, which was conducted via TANRIC platform. B. The basal expression of miR-221 in HK-2, 786-O, and Caki-1 cells were evaluated by qRT-PCR. C. The expression levels of miR-221 were detected by qRT-PCR after knockdown of TCL6 in 786-O cells. D. The expression level of miR-221 was further examined with or without upregulation of TCL6 using qRT-PCR. E. TCL6 contains two potential binding sites of miR-221. F. Relative luciferase activity in wild type of TCL6. The decrease in luciferase activity in the wild type indicated specific binding of miR-221 to its target site. Data are shown as means ± SD. *P<0.05.

The analysis of TANRIC platform showed that the level of miR-221 was significantly negatively correlated with TCL6 in renal cancer (Fig.5A). As shown in Fig.5B, miR-221 was elevated in RCC cells compared to HK2 cells. Silence of TCL6 increased the expression level of miR-221 in 786-O cells (Fig.5C), while overexpression of TCL6 decreased the expression level of miR-221 in Caki-1 cells (Fig.5D). According to miRcode, a map of putative microRNA target sites in the lncRNA, suggesting TCL6 is highly predicted to be a target of miR-221, and two potential binding sites were listed in Fig.5E. To investigate this hypothesis, the sequence of TCL6 was inserted into a pmirGLO-Luc vector. As shown in Fig.5F, the luciferase activity was suppressed when HEK293 cells were co-transfected with the pmirGLO-TCL6-Luc vector and miR-221 mimics, compared with those cells co-transfected with the pmirGLO-TCL6-Luc vector and negative control mimics. These results suggest that miR-221 directly binds to TCL6.

## Discussion

RCC is known to be a heterogeneous group of cancers with disparate genetic and molecular alterations underlying the various histological subtypes with no effective therapeutic options. In this study, we found the expression deficiency of TCL6 (located on chromosome 14) was observed in RCC tissues through analysis of TCGA and GEO datasets, which was evidenced by multi-platform. Low level of TCL6 was associated with worse overall and disease-free survival of RCC patients. These findings were consistent with a previous study, TCL6 was reported to be negatively correlated with the T, N, M, and TNM stage and serve as an independent predictor via analysis of 71 pairs of RCC samples 19. Here, we demonstrated that TCL6 mainly distributed in the cytoplasm.

Further research is warranted into developing novel therapies in combination with standard chemotherapy regimens with greater efficacy than a single treatment. To further investigate whether TCL6 exerts synergistic cytotoxicity effects with PTX in RCC cells, we modified the expression level of TCL6 and analyzed its impacts on the cell viability and apoptosis of 786-O and Caki-1 cells. We demonstrate that inhibition of TCL6 reduces the cytotoxicity of PTX in RCC cells. Conversely, up-regulation of TCL6 is effective in sensitizing RCC response to PTX chemotherapy. Several functions have been identified for lncRNAs including the recruitment of chromatin-remodeling complexes to specific genomic loci that modify histone and RNA polymerase accessibility, miRNAs-sponge binding, and multiple epigenetic changes 20. microRNA sponge is the main function of lncRNA in tumor chemotherapy. Such as, HOXA11-AS promotes cisplatin resistance of human LUAD cells via sponging miR-454-3p 21. Linc00518 was demonstrated to decrease chemosensitivity to breast cancer cells through regulating the miR-199a 22 and UCA1 confers PTX resistance to ovarian cancer through silencing miR-129 23. Since LNCRNAs mainly act as modulators of other classes of RNAs, we thus focused on their sponging functions. Here, we found that down-regulation of TCL6 was accompanied by increasing the expression of miR-221, while overexpression of TCL6 inhibited the endogenous miR-221. A negative correlation between TCL6 and miR-221 in RCC tissues was confirmed by analysis of RNA-seq data derived from the TANRIC database. The previous study showed that elevated expression of miR-221 in tissues 24 and plasma 25 was associated with a poor prognosis in RCC patients. Moreover, inhibition of miR-221 resulted in significantly enhancing the therapeutic efficacy of PTX 26. Thus, a possible mechanism explaining the association between higher TCL6 expression and higher PTX-induced cell apoptotic rate, was the sponging effect exerted by this LNCRNA on miR-221, which was confirmed by the luciferase reporter assay. Besides, we will further analyze the function of TCL6 by using RCC cell lines in the same design in the future study.

Collectively, our study elaborated a novel TCL6/miR-221 regulatory axis underlying PTX sensitivity of RCC cells, providing a potential therapeutic target for RCC.

## Abbreviations

lncRNAs: long non-coding RNAs
PTX: paclitaxel
RCC: renal cancer cells
TCGA: The Cancer Genome Atlas
GEO: Gene Expression Omnibus
FISH: Fluorescence in situ hybridization
TCL6: T-cell leukemia lymphoma 6
RCC: Renal cell carcinoma
GEPIA: Gene Expression Profiling Interactive Analysis
TANRIC: The Atlas of Noncoding RNAs in Cancer
ATCC: American Type Culture Collection
PI: propidium iodide
